# Longevity and Success of Restorative Treatments: Knowledge of Dentists and Laypeople

**DOI:** 10.1111/eje.70130

**Published:** 2026-03-09

**Authors:** Brenda Antonow Nunes Da Silva, João Gabriel Silva Souza, Fernando Freitas Portella

**Affiliations:** ^1^ Institute of Health Sciences, School of Dentistry Feevale University Novo Hamburgo Rio Grande do Sul Brazil; ^2^ School of Dentistry and Postgraduate Program in Dentistry and Orthodontics University of Guarulhos Guarulhos São Paulo Brazil

**Keywords:** dentists, knowledge, longevity, oral rehabilitation, patients

## Abstract

**Objective:**

To describe the knowledge of dentists and laypeople regarding the longevity and success rate of various restorative treatments in dentistry.

**Methods:**

This observational cross‐sectional study was conducted in two phases. In phase 1, a guided interview assessing the longevity of various restorative treatments (including direct composite resin, ceramic veneers, indirect restorations, success of root canal treatments, and implant‐supported prosthesis) was administered to 20 dental surgeons. Based on the analysis of the responses from phase 1, an objective questionnaire was developed and applied online to a convenience sample of dentists and laypeople. A descriptive analysis of the responses was performed, and the data were compared with the existing literature. Additionally, the association between the characteristics of the dentists and their responses was examined.

**Results:**

Phase 1 revealed considerable disparity in the longevity values reported by professionals for the same treatments. This heterogeneity of responses was instrumental in formulating the answer choices for the objective questionnaire used in the next phase. In Phase 2, 212 dentists and 398 laypeople responded to the survey. Of the laypeople, 68.6% visited the dentist in the past year, and 11.1% based their responses on dentist guidance. At least 11.3% of professionals couldn't identify the best option for describing treatment success or longevity, and responses varied greatly. The percentage of responses aligned with reference literature were: 43.4% for the longevity of composite resin restorations, 60.8% for ceramic veneers, 62.3% for indirect restorations in posterior teeth, 67.9% for successful root canal treatments, and 53.3% for implant‐supported prostheses.

**Conclusion:**

There is variability in the responses, with a significant portion of dentists demonstrating difficulty in providing accurate answers on the topic, while a considerable proportion of laypeople expect dental procedures to last a lifetime.

## 1 Introduction

The demand for restorative and aesthetic dental treatments has been steadily increasing. Despite the continued global prevalence of dental caries and periodontal diseases, there is a clear shift toward preserving natural teeth throughout a patient's lifetime. This trend has sparked growing interest among both the general public and dental professionals, creating a demand for treatments that are not only affordable and aesthetic but also prioritize patient health, sustainability, and ethical practice [1].

In general, the outcome and longevity of restorative procedures and dental therapies depend on various factors, such as patient characteristics, habits, techniques, operator experience, and the materials used [2]. Many of these factors are often unknown to laypeople, who may attribute treatment failures primarily to the materials. Today, more than 60 years after the introduction of high‐copper amalgam restorations, which demonstrated predictability, and over 60 years since Buonocore's demonstration of enamel acid etching, triggering discoveries that enabled the development of adhesive restorations [3], parameters have been established to estimate the longevity and success rates of the treatments available to professionals [2].

Dentistry offers many restorative procedures, with scientific literature providing data on their longevity to assist in decision‐making and treatment planning. However, professionals may face challenges in accessing scientific articles, critically evaluating them, or even reading them due to a lack of proficiency in English [4]. Additionally, communicating these longevity data and success rates to patients is challenging, as the literature presents these data in relative numbers (e.g., annual failure rate, survival at a specific follow‐up), whereas laypeople and even many clinical professionals tend to interpret numerical information more easily when presented in absolute terms. For better professional‐patient communication, the best approach is to use natural frequencies rather than percentages and conditional probabilities [5].

Thus, there is a need to understand the knowledge of both professionals and the general population on this topic, as this can foster better dialogue and case management, align the expectations of professionals and patients, and contribute to improved dental treatment planning and decision‐making. Therefore, the objective of this study is to describe the knowledge of dental professionals and laypeople regarding the longevity (measured by annual failure rate or success/survival rate) and success rates of a range of dental treatments. To achieve this, two complementary specific objectives were defined. First, to identify the possible answers for different dental treatments, thereby providing a basis for the development of an objective questionnaire. Second, to use this objective questionnaire to assess the knowledge of dentists and laypeople on the topic.

## 2 Methods

To address the objectives, this study was planned in two stages. In the first stage (Phase 1), 20 professionals were interviewed about the longevity of certain treatments. Based on the responses from these professionals and a review of the scientific literature, an objective questionnaire was developed and electronically administered (Phase 2). The questionnaire was divided into one section for dentists and another for laypeople. For both phases, the research protocol was approved by the Ethics Committee of Feevale University, at southern Brazil (CAAE 63832422.4.0000.5348 and CAAE 75131823.0.0000.5348), and subjects sign a consent prior to participates.

### 2.1 Interviews With Professionals—Phase 1

Twenty dental surgeons from the metropolitan region of Porto Alegre/Brazil were interviewed. They were recruited through convenience sampling based on personal contacts of the researchers. To ensure diversity, professionals from different settings were invited. A total of 20 dentists were interviewed, with an average response time of 4 min. Among the professionals, five worked exclusively as university professors, five practiced in private clinics, five worked in both private practice and the public health system, and another five, in addition to private practice, also taught at universities. Participants were initially contacted via text message to invite their participation. Upon acceptance, participants indicated their preferred interview method (either in‐person or remote, via video call or phone) and received the informed consent form.

The interviews were guided by a structured questionnaire, created by the authors, which included open‐ended questions (Appendix S1) about the longevity and success rates of various treatments. These treatments comprised direct restorations, indirect restorations, ceramic veneers, endodontic treatment, and implant‐supported prostheses. At this stage, no predefined answer options were provided, allowing participants to respond freely in terms of annual failure rate, success rate, or any other format. The interviews, conducted by a single researcher (BANS), were audio‐recorded and transcribed for subsequent analysis. A word cloud was created to visualise the main reasons for treatment failure.

### 2.2 Online Survey With Professionals and Laypeople—Phase 2

The knowledge of professionals and laypeople regarding the longevity and success rate of dental treatments was assessed through an online survey. The results from interviews conducted in Phase 1 and relevant scientific literature on the topic [6, 7, 8, 9, 10, 11] guided the development of objective questions (Appendix S2) on the theme. The spectrum of responses from the initial qualitative phase informed the development of the structured answer choices for the subsequent objective questionnaire. The comprehension and adjustments to the questions were evaluated after administering the questionnaire to 10 volunteer professionals who responded to the instrument. The questionnaire, originally created for professionals, was subsequently modified to include questions and options that are more comprehensible for laypeople (Appendix S2). Similarly to the questionnaire for professionals, the one for laypeople underwent adjustments based on feedback from 10 lay participants during testing.

The online survey gathered data on specific dental treatments, participant demographics (age, education), professional background (experience, procedures performed), and the motivations for their answers (e.g., scientific literature, clinical experience, online information). The sample comprised of dentists and laypeople who had internet access and digital communication means, with eligibility criteria including all professionals over 18 years of age. In the questionnaire for laypeople, a question was included about whether they worked in any task related to dentistry; those cases where the participant answered affirmatively were excluded from the analysis. The study employed a convenience sampling method. The two questionnaires were made available via Google Forms. Participation invitations were disseminated through information technology, using public profiles on the Instagram platform. Additionally, researchers shared the links via WhatsApp groups and sent them individually to profiles of professionals identified through Instagram searches. Accurately estimating the reach of posts on Instagram is challenging; nevertheless, a total of 267 professionals and 117 laypeople were invited through a direct approach based on the researchers' logistical capacity. The study experienced a non‐response rate, as not all invited professionals agreed to participate. These collection efforts were repeated six times from November 2023 to January 2024.

The collected data, aggregated in the online Google Forms, were downloaded and converted into an Excel spreadsheet format for descriptive analysis. Responses from professionals regarding the longevity and success rate of treatments were dichotomously categorised based on their alignment with scientific literature. The following data were arbitrarily used as literature parameters for longevity and success rate: annual failure rate of 4.6% for anterior composite resin restorations [6] and of 2.5% for posterior composite resin restorations [8], 95.5% survival rate over 10 years for ceramic veneers on anterior teeth [7], 90% survival rate over 10 years for ceramic restorations on posterior teeth [9]. Success rate ranging from 75% to 95% for endodontic treatments [10] and 96% survival rate at 3 years follow‐up for ceramic crowns supported by implants [11]. Associations between professionals' characteristics and these responses were analysed at 5% significance using either the chi‐square test or Fisher's exact test. Also, the association between the basis for answering questions on treatment longevity and success and education was evaluated.

## 3 Results

### 3.1 Interviews With Professionals—Phase 1

Open‐ended questions were employed to collect initial data (e.g., ‘What is the longevity of composite resin restorations in posterior teeth?’). No specific response format was stipulated, such as requesting an annual failure rate or a survival rate over a defined follow‐up period. Consequently, the responses are presented exactly as provided by the participants, without any subsequent categorization, in‐depth analysis, or interpretation by the investigators. According to the participants, the longevity of direct composite veneers ranges from 2 to 15 years (with a commonly cited estimate of 10 years). For ceramic veneers, responses ranged from 7 to 30 years, with 10 years being the most cited estimate. Regarding composite resin restorations in posterior teeth, the most cited longevity was 5 years, ranging from 5 to 30 years. For full ceramic crowns in posterior teeth, the responses ranged from 10 to 30 years, with 10 years being the most prevalent estimate. The clinical success rate of endodontic treatments varied from 70% to 95%, with an estimated average of 90%. Finally, the clinical success rate of implant installation ranged from 70% to 98%, with an estimated average of 95%. Regarding factors associated with the longevity and success of treatments, key aspects included patient habits, occlusal factors, materials and techniques used (especially for ceramics), and regular dental check‐ups, as represented on word cloud in Figure 1.

**FIGURE 1:**
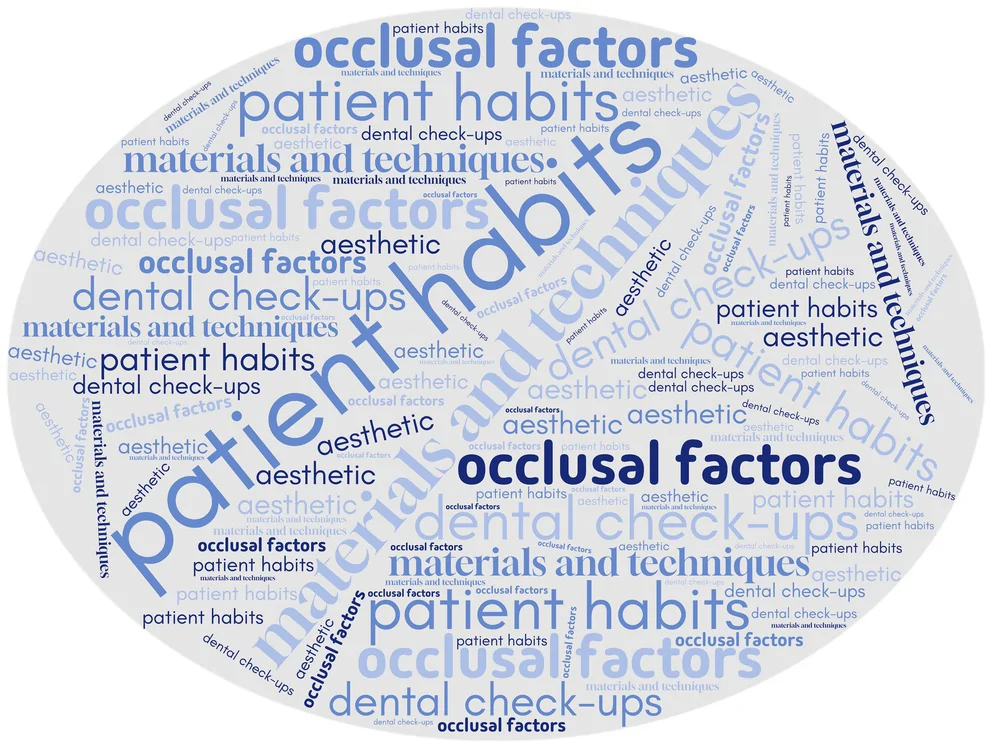
Representative word cloud of the principal associated factors of failure for restorative treatments cited by interviewed professionals.

### 3.2 Online Survey With Professionals and Laypeople—Phase 2

The recruitment strategy, which combined an open invitation on social media with direct personal invitations, yielded a final sample of 212 dentists and 440 laypersons. Among the lay respondents, 42 had occupations related to dental health services and were excluded from the analysis. Table 1 presents the characterisation of the participants.

**TABLE 1:** Characteristics of professionals and laypersons who responded to the online survey.

Characteristic	Data
Professionals	*n* = 212
Age in years (mean ± standard deviation)	36.7 ± 9.0
Years since graduation (mean ± standard deviation)	12.0 ± 9.1
*Education*	
Specialisation	142 (67.0%)
Master's degree	71 (33.5%)
PhD	42 (19.8%)
*Postgraduate areas of knowledge (top five cited)*	
Implantology	44 (20.8%)
Prosthodontics	43 (20.3%)
Orthodontics	35 (16.5%)
Periodontology	27 (12.7%)
Public Health	23 (10.8%)
*Professional activities*	
Clinical practice in private office or clinic	187 (88.2%)
Clinical practice in public health system	36 (17.0%)
Teaching (undergraduate, specialisation, master's and doctorate)	60 (28.3%)
Manager in public health services	5 (2.4%)
Radiologist	2 (0.9%)
None of the above	5 (2.4%)
*Procedures performed at least twice a week*	
Do not attend patients	7 (3.3%)
Direct composite resin restorations in posterior teeth	142 (67.0%)
Indirect composite resin restorations in posterior teeth	30 (14.2%)
All‐ceramic or partial ceramic crowns in posterior teeth	63 (29.7%)
Composite resin veneers	55 (25.9%)
Ceramic laminates	36 (17.0%)
Root canal treatments	39 (18.4%)
Implant installation surgery	48 (22.6%)
Periodontal treatments	93 (43.9%)
Orthodontic treatments	33 (15.6%)
Temporomandibular dysfunction treatments	16 (7.5%)
Facial harmonisation	21 (9.9%)
Extractions	105 (49.5%)
*Basis for answering questions on treatment longevity and success*	
Scientific literature	86 (40.6%)
Clinical experience	177 (83.5%)
Courses, lectures, or internet sources	65 (30.7%)
**Laypersons**	***n* = 398**
Age in years (mean ± standard deviation)	32.0 ± 12.2
*Education*	
Elementary school	29 (7.3%)
High school	158 (39.7%)
Undergraduate	169 (42.5%)
Master's and/or PhD	42 (10.6%)
Visited dentist in the last year	273 (68.6%)
*Treatments received*	
Don't remember or have not received any of these treatments	95 (23.9%)
Composite resin restoration in posterior tooth	271 (68.1%)
Resin veneer	28 (7.0%)
Ceramic veneer	11 (2.8%)
Indirect ceramic restorations	29 (7.3%)
Root canal treatment	160 (40.2%)
Dental implant	49 (12.3%)
*Basis for answering questions on treatment longevity and success*	
Guidance from dentists	44 (11.1%)
Common sense or personal expectations	367 (92.2%)
Internet	12 (3.0%)

The responses from dentists and laypeople to each of the questions about the longevity and success of treatments are described in Figure 2. For all treatments, both among professionals and laypeople, there is a variation in responses. Among dentists, at least 11.3% of them either did not know or felt uncomfortable in pointing out the best alternative that would describe the longevity or success of the treatment. The proportion of responses aligning with the evidence from the reference literature [6, 7, 8, 9, 10, 11] was 92 (43.4%) for composite resin restoration longevity, 129 (60.8%) for ceramic laminates, 132 (62.3%) for posterior indirect restorations, 144 (67.9%) for root canal treatment success, and 113 (53.3%) for implant‐supported prostheses.

**FIGURE 2:**
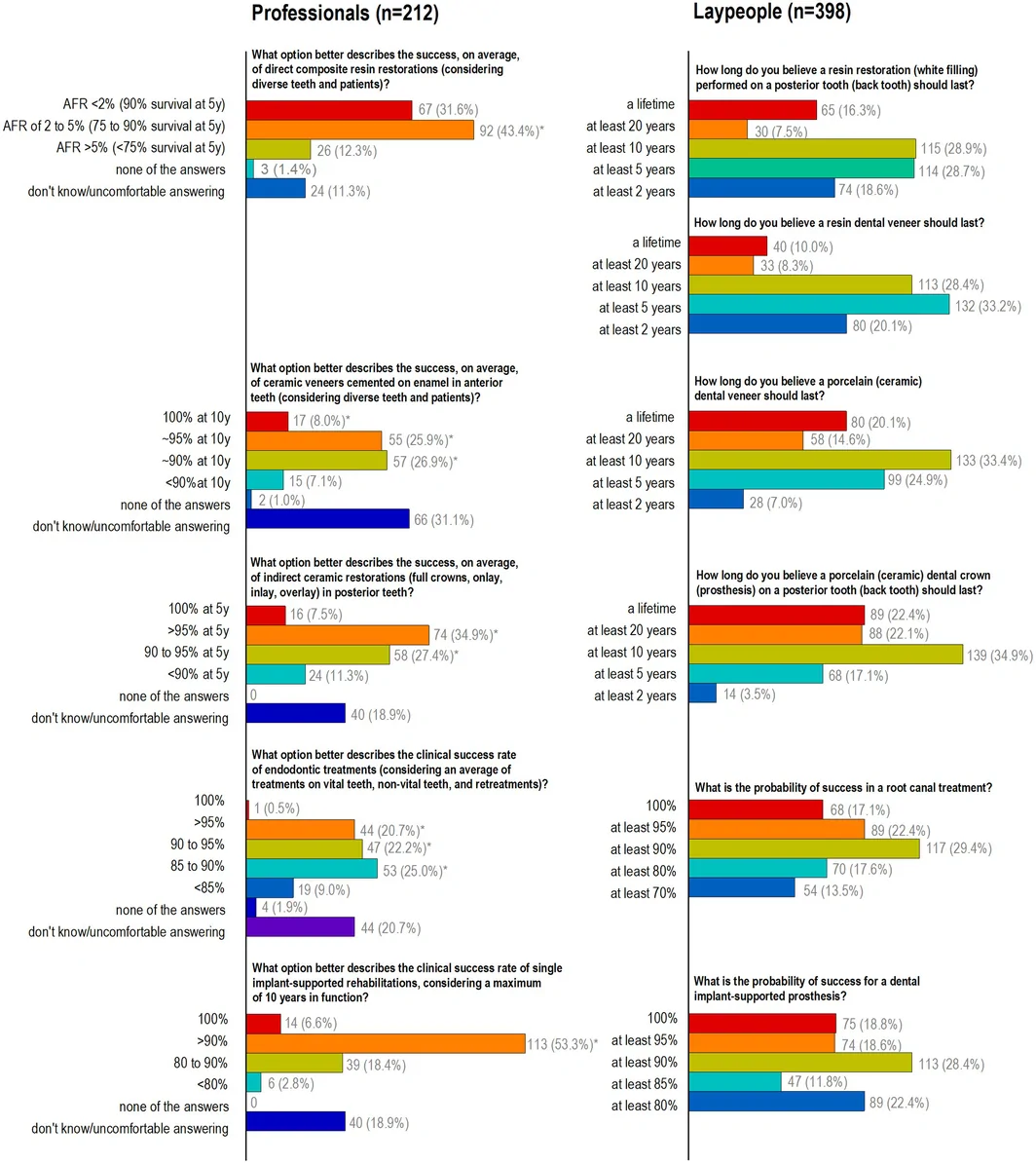
Responses from dentists and laypeople regarding the longevity and success of rehabilitative treatments. *Responses approximating scientific literature [6, 7, 8, 9, 10, 11]. AFR, Annual failure rate. The terms in the figure are based on the translated version of the questionnaire that was presented to participants. The original Portuguese version is available in Appendix S2.

The dentists were requested to specify the knowledge sources they relied on to respond to inquiries regarding the durability of the procedures referenced in the study. The results showed that 83.5% relied on their clinical experience, 40.6% on scientific literature, and 30.7% on knowledge from courses, lectures, or the internet. Likewise, the laypersons were inquired about the knowledge base they utilized to respond to the questions. Results indicated that 92.2% of the sample relied on common sense or their own expectations of procedure longevity, 3% on internet‐based knowledge, and 11.1% on advice given by dentists.

The association between professionals' characteristics and the alignment of their responses with the literature was evaluated (Table 2). Among the characteristics associated with responding more in line with the literature for some of the questions were: having completed postgraduate education in a rehabilitative field (such as prosthodontics, restorative dentistry, implantology, dental materials), performing rehabilitative procedures at least twice a week, and practicing in a private office. Those who based their responses solely on clinical experience or who reported working in public health services showed more responses inconsistent with the literature. Additionally, participants who had Master's or doctorate degrees were associated with responding based on scientific articles, while respondents with specialisation postgraduate education increased the likelihood of responding based on clinical experience.

**TABLE 2:** Association between the professionals' characteristics and the consistency of their responses with literature.

Characteristics	Treatment (responses aligned with the literature)				
	Direct composite resin (43.4%)	Ceramic veneers (60.8%)	Ceramic on posterior teeth (62.3%)	Root canal treatment (67.9%)	Single‐unit implant‐supported rehabilitations (53.3%)
Master's and/or PhD	ns	ns	ns	ns	ns
Specialisation	ns	ns	ns	ns	ns
Postgraduate studies in a rehabilitative area (prosthodontics, operative dentistry, implant dentistry, dental materials)	ns	✓	✓	ns	✓
Clinical practice in private office or clinic	ns		ns	ns	✓
Clinical practice in public health system	ns	x	ns	ns	ns
Teaching (undergraduate, specialisation, master's, and doctorate courses)	ns	ns	ns	Ns	ns
Performed restorative treatments at least twice a week	✓	ns	ns	ns	ns
Respond to the questions only based on clinical experience	ns	x	ns	ns	Ns

Abbreviations: ✓, responses aligned with the literature; ns, non‐significant association; x, responses inconsistent with the literature.

## 4 Discussion

A wide variation in reported knowledge was observed both among professionals, both in phases 1 and 2, and laypeople. A noteworthy observation is the significant proportion of dentists who responded that they did not know or felt uncomfortable with some of the questions regarding longevity in phase 2 of the study.

In the initial phase involving 20 dentists, it was observed that responses regarding the longevity of restorative treatments exhibited considerable variability, yet generally aligned with existing scientific literature [6, 7, 8, 9, 10, 11]. Overall, the reasons dentists attributed to these failures corresponded to findings in the scientific literature [6, 7, 8, 9, 10, 11], such as technique used, operator skills, secondary caries, fractures, occlusion, patient habits (diet, hygiene, socio‐economic factors), and the location of the dental element.

To the authors' knowledge, this study is original in providing information on the knowledge of dentists and laypersons regarding the longevity of rehabilitative treatments. Investigating the longevity and success of dental treatments is challenging. It requires studies with large sample sizes and adequate follow‐up periods. The diverse methodologies employed in these studies often yield heterogeneous results [8, 12, 13, 14], which can be difficult for clinicians to interpret. This difficulty is further exacerbated by the fact that outcomes are typically reported as relative metrics (e.g., annual failure rates or survival/success rates over specific time intervals) rather than absolute numbers. Consequently, these challenges may also translate into difficulties for professionals when communicating prognostic information to patients in an easily understandable manner. There is no definitive value for the longevity and success of any treatment in health services. Thus, professionals have approximate parameters to guide their communication with patients. These are some of the hypotheses proposed by the researchers. However, the current lack of comprehensive studies on this specific topic limits the possibility of direct comparisons with other studies in the literature.

When comparing the responses in Phase 2 of the study, conducted quantitatively with 212 dentists and 398 laypeople, several key points emerge. A significant portion of laypeople (average of 17.4%) expect dental procedures to last a lifetime without considering potential failures. Moreover, the fact that 68.6% of the sample visited a dentist in the last year and only 11.1% of their knowledge comes from dentist‐provided guidance suggests that the longevity of treatments may not be discussed as thoroughly as it should be between professionals and patients. In relation to specific questions regarding the success of direct composite resin restorations, 43.4% of professionals indicated an annual failure rate between 2% and 5%, aligning closely with scientific literature where the annual failure rate for posterior composite resin restorations is 2.5% [8] and for composite resin veneers is 4.6% [6]. However, 31.6% of dentists responded that the failure rate would be below 2%, which contradicts scientific literature and could potentially raise patient expectations regarding the durability of these procedures. This discrepancy is evident in the layperson sample, where 16.3% expected a composite resin restoration in a posterior tooth to last a lifetime, and 10% expected the same for composite resin veneers.

Evaluating the durability of ceramic veneers, it is important to note that 60.8% of the respondents provided answers consistent with scientific literature, citing success rates of approximately 90%, 95%, and 100% after 10 years of use. Scientific literature indicates that the longevity of ceramic veneers is around 95.5% over 10 years [7]. However, what stands out in this question is that 31.1% of professionals reported not knowing or feeling uncomfortable to respond, which was the most frequently selected isolated alternative. It is also important to highlight that 20% of laypeople believed ceramic veneers should last a lifetime, and 14.6% believed they should last at least 20 years, totaling 34.6% of laypeople with expectations above the average elucidated by the literature regarding veneers.

Regarding indirect ceramic restorations on posterior teeth, literature suggests a success rate of 90%–95% or over 95% after 5 years, with 62.3% of responses aligning with this. This corresponds well with findings in the literature which indicate a success rate of over 90% after 5 years of follow‐up [9]. Once again, a significant portion of dentists (18.9%) indicated not knowing the answer. It was also observed that 34.9% of laypeople had expectations aligned with reality. However, 22.4% and 22.1% expected these restorations to last a lifetime or at least 20 years, respectively. Together, 44.5% of laypeople expect significantly longer durability than what scientific literature shows. This mismatch may be associated with the 7.5% of dentists who also do not expect failures within 5 years, indicating a 100% success rate for this timeframe.

In the survey about the clinical success of root canal treatments, around 67.9% of the responses were consistent with scientific literature, categorising the success rates as between 85% and 90%, between 90% and 95%, and above 95%. These responses are consistent with studies reporting success rates ranging from 75% to 95% for root canal treatments of necrosis and pulpitis, respectively [10]. Once again, it is notable that 20.7% of professionals did not know or did not feel comfortable responding to this question. Generally, laypeople's responses were also aligned with the literature, although some had high expectations; 17.1% expected a success rate of 100%, indicating no chances for treatment failure in endodontics.

In the question regarding the clinical success of single‐unit implant‐supported rehabilitations, only the option stating success rates above 90% was considered approximate to scientific literature. This option was chosen by 53.3% of the total sample of dentists, aligning with a reported success rate of 96% for implant‐supported crowns after 3 years of follow‐up [11]. It is important to note that a significant portion of dentists (18.9%) reported not knowing how to respond to this question, and 6.6% indicated a 100% success rate, suggesting no chance for failure across all cases for all patients. Laypeople's responses were generally aligned with the literature, with the majority expecting success rates of at least 90% or 80% of cases. Additionally, 18.8% of laypeople, like professionals, expected a 100% success rate across cases.

In this study, aiming to assess the professionals' knowledge regarding the estimation of longevity and success of treatments, the research questionnaire presented some alternatives with values close to a references [6, 7, 8, 9, 10, 11] arbitrarily established by the authors, which were used in the analyses. Different answers from these are not necessarily incorrect values. Nonetheless, a significant number of participants indicated that there were no suitable options among those presented or even marked that they did not know or felt uncomfortable responding. The lack of this knowledge regarding longevity and success rates could lead to confusion for both dentists and patients when choosing dental treatment plans, potentially resulting in conflicts between both parties when patients feel aggrieved—such as the failure of a dental procedure in a shorter time than the patient expected, and also not supported by literature. This obstacle in communication between professionals and patients could jeopardise evidence‐based practice. Although professionals may be inclined to obtain scientific information, they face challenges in accessing full articles and critically evaluating them [4].

There was no statistically significant difference between having completed postgraduate education (master/doctorate or specialisation) and aligning responses closer to the scientific literature. Being a teacher or exclusively dedicating oneself to clinical dental practice also did not correlate with greater or lesser alignment with the literature in responses. However, when analysing the influence of the postgraduate area, individuals specialising in Restorative Dentistry, Prosthodontics, and/or Implantology showed a higher probability of aligning with the literature in the questions posed in this study. Similarly, participants who reported performing rehabilitation procedures in clinical settings at least twice a week had a higher probability of answering correctly regarding the longevity of composite resin restorations. This is easily understood, considering that the questions are related to the study area of these specialties. It is positive as it indicates that professionals specialised in the rehabilitation field are more likely to have knowledge about the longevity of the procedures they perform. It also makes sense that professionals performing these procedures frequently are more likely to answer correctly, as frequent execution implies a deeper understanding of the factors involved in the treatment, including its longevity. The statistical analysis further revealed that participants working in the public health system showed a greater tendency to answer questions related to the longevity of ceramic veneers incorrectly. This aligns with their reality in Brazil, as ceramic veneers are not typically performed in the public health system. On the other hand, those working in private practices tended to answer correctly more often in questions concerning the durability of prostheses supported by implants, likely due to performing this procedure more frequently in their practices.

It was observed that participants with master's and/or doctorate degrees were more inclined to base their responses on scientific articles, which naturally indicates a higher likelihood of answering correctly. In contrast, respondents with specialisations had a greater probability of answering based on clinical experience, which, in turn, showed a tendency to provide incorrect responses. This result may indicate a trend of specialisation courses to focus on clinical practice or fail on learning to read and interpret research papers.

Based on the findings of this study, and considering the limitations to extrapolating the results, such as the convenience sample, there is a recognised need and urgency for professionals to update their knowledge in this area through courses, seminars, and primarily studying the available scientific literature related to the longevity and success of restorative treatments. It is crucial to structure dental education to emphasise these topics in dental schools at all levels of training, undergraduate or postgraduate.

## 5 Conclusion

At least 10% of the dentists demonstrated difficulty in providing accurate answers regarding the longevity and success of the investigated dental treatments. The proportion of professionals whose responses were consistent with the literature was 43.4% for composite resin restoration longevity, 60.8% for ceramic laminates, 62.3% for posterior indirect restorations, 67.9% for root canal treatment success, and 53.3% for implant‐supported prostheses. Furthermore, a considerable proportion of laypeople, also exceeding 10%, held the expectation that dental procedures would last a lifetime.

## Funding

The authors have nothing to report.

## Conflicts of Interest

The authors declare no conflicts of interest.

## Supporting information


**Appendix S1:** Structured questionnaire used in Phase 1.

**Appendix S2:** Objective questions used in Phase 2.

## Data Availability

The data that support the findings of this study are available on request from the corresponding author. The data are not publicly available due to privacy or ethical restrictions.
