# Supplementary material for: Longevity and Success of Restorative Treatments: Knowledge of Dentists and Laypeople
Source: Eur J Dent Educ. 2026 Mar 9;30(4):1921–8. doi: 10.1111/eje.70130 (PMC13686682; doi:10.1111/eje.70130)
Supplement: Supplementary file 1 [file EJE-30-1921-s001.pdf]

Longevidade de tratamentos restauradores

Olá, que bom te ver aqui!

Meu nome é Brenda e gostaria da sua ajuda respondendo a um questionário para o meu TCC, na Universidade Feevale. Estou orientada pelo professor Fernando Portella.

Em menos de 3 minutos você termina.

Este estudo foi aprovado em Comitê de Ética (link aqui).

brendantonow@gmail.com Alternar conta
Não compartilhado
Rascunho salvo.

* Indica uma pergunta obrigatória

Ano de nascimento (registrar somente o ano) *

Sua resposta

Ano de formatura (registrar somente o ano) *

Sua resposta

Formação (marque todas que possui) *

- [ ] Graduação
- [ ] Especialização
- [ ] Mestrado
- [ ] Doutorado

Área da pós-graduação (seja especialização, seja mestrado, seja doutorado - marcar todas que possui) *

- [ ] Não realizei pós-graduação
- [ ] Acupuntura
- [ ] Cirurgia e Traumatologia Buco Maxilo Faciais
- [ ] Dentistica
- [ ] Disfunção Temporomandibular e dor Orofacial
- [ ] Endodontia
- [ ] Estomatologia
- [ ] Harmonizacao Orofacial
- [ ] Homeopatia
- [ ] Implantodontia
- [ ] Materiais Dentários
- [ ] Odontogeriatria
- [ ] Odontologia do Esporte
- [ ] Odontologia do Trabalho
- [ ] Odontologia Legal
- [ ] Odontologia Para Pacientes Com Necessidades Especiais
- [ ] Odontopediatria
- [ ] Ortodontia
- [ ] Ortopedia Funcional dos Maxilares
- [ ] Patologia Oral e Maxilo Facial
- [ ] Periodontia
- [ ] Prótese Buco Maxilo Facial
- [ ] Prótese Dentária
- [ ] Radiologia Odontologica e Imaginologia
- [ ] Saúde Coletiva
- [ ] Outro

Atuação profissional (marcar todas em que atua) *

- [ ] Atendimento clínico em consultório ou clínica privada
- [ ] Atendimento clínico no SUS
- [ ] Docência (considerar apenas docência em cursos de graduação, especialização, mestrado e doutorado)
- [ ] Gestor em serviços de saúde pública
- [ ] Atendimento de pacientes e/ou elaboração de laudos em clínicas de Radiologia
- [ ] Não atuo em nenhum dos campos descritos acima

Estado onde reside *

- ( ) Rio Grande do Sul
- (●) Outro

Dentre os procedimentos abaixo, marque aqueles que você realiza ao menos 2x na semana (em média): *

- [ ] não realizo atendimento de pacientes
- [ ] restaurações diretas em resina composta em dentes posteriores
- [ ] restaurações indiretas em resina composta em dentes posteriores
- [ ] coroas totais ou restaurações parciais em cerâmica em dentes posteriores
- [ ] facetas em resina composta
- [ ] laminados cerâmicos
- [ ] tratamentos endodônticos
- [ ] cirurgia de instalação de implantes
- [ ] tratamentos periodontais
- [ ] tratamentos ortodônticos
- [ ] tratamentos de disfunção têmporo-mandibular
- [ ] harmonização facial
- [ ] extrações

Qual alternativa melhor descreve o sucesso, em média, de restaurações diretas em resina composta (considerando dentes e pacientes com características diversas)? *

- ( ) taxa de falha anual inferior a 2% (longevidade de 90% após 5 anos em função)
- ( ) taxa de falha anual entre 2% e 5% (longevidade entre 75% e 90% após 5 anos em função)
- ( ) taxa de falha anual superior a 5% (longevidade inferior a 75% após 5 anos em função)
- ( ) nenhuma das alternativas apresenta valores adequados
- ( ) não sei ou não me sinto confortável em responder

Qual alternativa melhor descreve o sucesso, em média, de laminados cerâmicos cimentados em esmalte em dentes anteriores (considerando dentes e pacientes com características diversas)? *

- ( ) aproximadamente 100% após 10 anos em função
- ( ) aproximadamente 95% após 10 anos em função
- ( ) aproximadamente 90% após 10 anos em função
- ( ) menos de 90% após 10 anos em função
- ( ) nenhuma das alternativas apresenta valores adequados
- ( ) não sei ou não me sinto confortável em responder

Qual alternativa melhor descreve o sucesso, em média, de restaurações indiretas (coroas totais, onlay, inlay, overlay) cerâmicas em dentes posteriores? *

- ◯ 100% após 5 anos em função
- ◯ superior a 95% após 5 anos em função
- ◯ entre 90 e 95% após 5 anos em função
- ◯ inferior a 90% após 5 anos em função
- ◯ nenhuma das alternativas apresenta valores adequados
- ◯ não sei ou não me sinto confortável em responder

Qual alternativa melhor descreve a taxa de sucesso clínico de tratamentos endodônticos (considerando uma média de tratamentos em dentes vitais, dentes não vitais e retratamentos)? *

- ◯ 100%
- ◯ acima de 95%
- ◯ entre 90 e 95%
- ◯ entre 85 e 90%
- ◯ inferior a 85%
- ◯ nenhuma das alternativas apresenta valores adequados
- ◯ não sei ou não me sinto confortável em responder

Qual alternativa melhor descreve a taxa de sucesso clínico de reabilitações unitárias sobre implantes, considerando um tempo de no máximo 10 anos em função? *

- ◯ 100%
- ◯ acima de 90%
- ◯ entre 80 e 90%
- ◯ inferior a 80%
- ◯ nenhuma das alternativas apresenta valores adequados
- ◯ não sei ou não me sinto confortável em responder

Para responder às questões anteriores você teve como base (pode marcar mais de uma) *

- ☐ literatura científica
- ☐ minha experiência clínica
- ☐ informações recebidas em cursos, palestras ou internet

Durabilidade e sucesso de tratamentos odontológicos

Olá, que bom te ver aqui!

Meu nome é Brenda e gostaria da sua ajuda respondendo a um questionário para o meu TCC, na Universidade Feevale. Estou orientada pelo professor Fernando Portella.

Em menos de 3 minutos você termina.

Este estudo foi aprovado em Comitê de Ética (link aqui).

Você trabalha ou tem formação em Odontologia (graduação, técnico ou auxiliar)? *

- ○ sim
- ○ não

Ano de nascimento (registrar somente o ano) *

Texto de resposta curta

Escolaridade (considere o maior nível concluído) *

- ☐ Alfabetizado
- ☐ Ensino fundamental
- ☐ Ensino médio
- ☐ Ensino superior
- ☐ Mestrado e/ou doutorado

Em que ano foi a sua última consulta ao dentista? *

- ○ 2023
- ○ 2022
- ○ 2021
- ○ 2020
- ○ 2019
- ○ 2018 ou anterior
- ○ nunca fui ao dentista

Dentre os tratamentos listados abaixo, marque aqueles que você lembra já ter recebido: *

- ☐ restauração (obturação) em dentes posteriores (do fundo)
- ☐ facetas (lente dental) em resina
- ☐ lentes dentais em cerâmica (porcelana)
- ☐ próteses em cerâmica (porcelana) em dentes posteriores (do fundo)
- ☐ tratamento de canal
- ☐ implante dentário
- ☐ não lembro ou não recebi nenhum desses tratamentos

Quanto tempo você acredita que deva durar uma restauração (obturação) em resina (massinha branca) realizada em um dente posterior (do fundo)? *

- ○ ao menos 2 anos
- ○ ao menos 5 anos
- ○ ao menos 10 anos
- ○ ao menos 20 anos
- ○ a vida toda

Quanto tempo você acredita que deva durar uma lente dental (faceta) em resina?

- ○ ao menos 2 anos
- ○ ao menos 5 anos
- ○ ao menos 10 anos
- ○ ao menos 20 anos
- ○ a vida toda

Quanto tempo você acredita que deva durar uma lente dental em porcelana (cerâmica)? *

- ○ ao menos 2 anos
- ○ ao menos 5 anos
- ○ ao menos 10 anos
- ○ ao menos 20 anos
- ○ a vida toda

Quanto tempo você acredita que deve durar uma prótese (coroa) em porcelana (cerâmica) em um dente posterior (do fundo)? *

- ○ ao menos 2 anos
- ○ ao menos 5 anos
- ○ ao menos 10 anos
- ○ ao menos 20 anos
- ○ a vida toda

Qual a probabilidade de sucesso em um tratamento de canal? *

- ○ ao menos 70%
- ○ ao menos 80%
- ○ ao menos 90%
- ○ ao menos 95%
- ○ 100%

Qual a probabilidade de uma prótese sobre implante dentário ter sucesso? *

- ○ ao menos 80%
- ○ ao menos 85%
- ○ ao menos 90%
- ○ ao menos 95%
- ○ 100%

Para responder às questões anteriores você teve como base (pode marcar mais de uma):

- ☐ orientações dadas por dentistas
- ☐ senso comum ou expectativas minhas
- ☐ informações da internet
