# Supplementary material for: Longevity and Success of Restorative Treatments: Knowledge of Dentists and Laypeople
Source: Eur J Dent Educ. 2026 Mar 9;30(4):1921–8. doi: 10.1111/eje.70130 (PMC13686682; doi:10.1111/eje.70130)
Supplement: Supplementary file 2 [file EJE-30-1921-s002.pdf]

Instrumento de Pesquisa<br>CONHECIMENTO DOS PROFISSIONAIS SOBRE A LONGEVIDADE DOS TRATAMENTOS RESTAURADORES	
Código participante:	Gênero:
Tipo de atuação profissional (pública, privada, docência):	
Ano de nascimento:	Ano de formatura:
Formação acadêmica (especialização, mestrado/doutorado):	
Se docente, trabalha com pesquisa:	
Procedimentos que mais realiza no consultório:	
**Perguntas norteadoras:**	
Qual a longevidade de facetas em resina composta direta?	
Qual a longevidade de facetas em cerâmica?	
Qual a longevidade de restaurações em resina composta em dentes	
posteriores?	
Qual a longevidade de reabilitações com coroas totais cerâmicas em dentes	
posteriores?	
Qual a taxa de sucesso clínico de tratamentos endodônticos?	
Qual a taxa de sucesso clinico da instalação de implantes?	
**Transcrição da entrevista:**	
